# The Delay in the Licensing of Protozoal Vaccines: A Comparative History

**DOI:** 10.3389/fimmu.2020.00204

**Published:** 2020-03-06

**Authors:** Clarisa Beatriz Palatnik-de-Sousa, Dirlei Nico

**Affiliations:** ^1^Institute of Microbiology Paulo de Góes, Federal University of Rio de Janeiro, Rio de Janeiro, Brazil; ^2^Institute for Research in Immunology, Faculty of Medicine, University of São Paulo, São Paulo, Brazil

**Keywords:** anti-protozoal vaccines, Malaria vaccines, *Leishmania* vaccines, vaccine development, vaccine evolution

## Abstract

Although viruses and bacteria have been known as agents of diseases since 1546, 250 years went by until the first vaccines against these pathogens were developed (1796 and 1800s). In contrast, Malaria, which is a protozoan-neglected disease, has been known since the 5th century BCE and, despite 2,500 years having passed since then, no human vaccine has yet been licensed for Malaria. Additionally, no modern human vaccine is currently licensed against Visceral or Cutaneous leishmaniasis. Vaccination against Malaria evolved from the inoculation of irradiated sporozoites through the bite of Anopheles mosquitoes in 1930's, which failed to give protection, to the use of controlled human Malaria infection (CHMI) provoked by live sporozoites of *Plasmodium falciparum* and curtailed with specific chemotherapy since 1940's. Although the use of CHMI for vaccination was relatively efficacious, it has some ethical limitations and was substituted by the use of injected recombinant vaccines expressing the main antigens of the parasite cycle, starting in 1980. Pre-erythrocytic (PEV), Blood stage (BSV), transmission-blocking (TBV), antitoxic (AT), and pregnancy-associated Malaria vaccines are under development. Currently, the RTS,S-PEV vaccine, based on the circumsporozoite protein, is the only one that has arrived at the Phase III trial stage. The “R” stands for the central repeat region of *Plasmodium (P.) falciparum* circumsporozoite protein (CSP); the “T” for the T-cell epitopes of the CSP; and the “**S**” for hepatitis B surface antigen (HBsAg). In Africa, this latter vaccine achieved only 36.7% vaccine efficacy (VE) in 5–7 years old children and was associated with an increase in clinical cases in one assay. Therefore, in spite of 35 years of research, there is no currently licensed vaccine against Malaria. In contrast, more progress has been achieved regarding prevention of leishmaniasis by vaccine, which also started with the use of live vaccines. For ethical reasons, these were substituted by second-generation subunit or recombinant DNA and protein vaccines. Currently, there is one live vaccine for humans licensed in Uzbekistan, and four licensed veterinary vaccines against visceral leishmaniasis: Leishmune® (76–80% VE) and CaniLeish® (68.4% VE), which give protection against strong endpoints (severe disease and deaths under natural conditions), and, under less severe endpoints (parasitologically and PCR-positive cases), Leishtec® developed 71.4% VE in a low infective pressure area but only 35.7% VE and transient protection in a high infective pressure area, while Letifend® promoted 72% VE. A human recombinant vaccine based on the Nucleoside hydrolase NH36 of *Leishmania (L.) donovani*, the main antigen of the Leishmune® vaccine, and the sterol 24-c-methyltransferase (SMT) from *L. (L.) infantum* has reached the Phase I clinical trial phase but has not yet been licensed against the disease. This review describes the history of vaccine development and is focused on licensed formulations that have been used in preventive medicine. Special attention has been given to the delay in the development and licensing of human vaccines against Protozoan infections, which show high incidence worldwide and still remain severe threats to Public Health.

## Vaccine Evolution

Vaccines are antigenic preparations used for the induction or enhancement of a pre-existing immunity of animals and humans to prevent or cure a disease or to promote the control of its transmission. They are the best cost-effective tools for controlling infectious diseases, and recently, their use has been extended with success to the immunotherapy of cancer based on unleashing immune checkpoints. Vaccines and sanitation were the two largest contributions to public health in the last two centuries. They both contributed to reducing the morbidity and mortality of infectious diseases (1, 2).

One of the first strategies to prevent diseases was to cause a mild primary infection with the original pathogen that would cure spontaneously and would provide protection against the full disease in the future (2). In the case of smallpox, however, this process, called variolation, caused the disease itself in 3% of the vaccinated individuals. Live vaccines were also used against *Leishmania* infections in the former URSS and Middle East (3). However, this practice was discontinued because of the recent increase in the population of immunocompromised subjects, in which live or attenuated vaccines could revert to virulence.

The second step in the evolution of vaccines consisted of using a live pathogen that causes an analogous disease in another species in order to cause heterologous cross-protection (cowpox) but not the disease. Afterward, the use of the homologous live pathogens that underwent attenuation started in the 1960's with the Sabin vaccine against poliomyelitis. This was followed by the first-generation vaccines, comprising dead or inactivated microorganisms or lysates (pertussis, cholera Typhus, paratyphus, Influenza, rabies, and anti-poliomyelitis Salk) or inactivated exotoxins (diphtheria and tetanus), which are immunogenic but cannot revert to virulence. The second-generation vaccines against bacteria include native pathogen molecules (anti-pneumococcus and anti-meningitis) and recombinant proteins (anti-Hepatitis B) (2), while against *Leishmania* they include mutated parasites causing abortive infection in mammals, non-pathogenic bacteria expressing *Leishmania* genes, and defined synthetic or recombinant subunits and partially purified fractions of the parasite (4). The third-generation DNA vaccines are composed of plasmids codifying for the most immunogenic proteins of the pathogen (4), and the most immunogenic epitope regions of the pathogen recently started to be combined multi-epitope synthetic vaccines (5, 6).

However, as vaccines of increased safety were developed, they lost their efficacy attributed to the use of whole pathogens or native toxin components corresponding to pathogen-associated molecular patterns (PAMPS) (7). To compensate for that loss and maintain efficacy and safety, purified or synthetic modern vaccines also included adjuvants (7).

## History of Vaccine Development

The habit of exposing an individual to a natural infection in order to render him immune and protect him from future diseases has a long history, for instance with plague, variolation, and leishmanization (2, 8, 9). The first record of variolation comes from China, where dried pustules from patients with smallpox were blown into the noses of healthy people or scratched into their skins to induce a mild self-resolved infection that would give future protection against the severe form of the disease (10). This practice was introduced into the Ottoman Empire by caravans from Asia and Africa in the early 1670s (11). Later, in 1796, Edward Jenner, a British physician who was himself variolized, inoculated material obtained from lesions of cowpox in an 8-year-old child, who showed protection when challenged with material obtained from pustules of smallpox infection. Jenner did not know at that time that he was inoculating a virus. The work of Jenner then reproduced variolation but with the cowpox virus, generating mild lesions that give high cross-protection (10, 11). The strong merit of Jenner's work was, however, not the invention of the Variola vaccine but the universalization of its use. He actually convinced George Washington to vaccinate the USA army. To date, the biological origin of vaccinia virus is uncertain, and it has been suggested that a horsepox-like pigpox, or even smallpox, virus was an ancestor (12). The success of this practice was evident, and massive vaccination in all countries was performed under a special intensified campaign promoted by the WHO in 1966. In 1977, smallpox was considered eradicated (8). Rinderpest was the second global lethal disease caused by a virus and was also eradicated by vaccination (13). It killed calves and provoked millions of human deaths related to the famines that it induced. In 1980, an attenuated virus vaccine began to be applied against rinderpest (13) and its vaccine-induced eradication was announced in 2011 (14).

## The Evolution of Bacterial and Virus Vaccine Technology

While Jenner was responsible for the establishment of the worldwide practice of preventive vaccination, scientific knowledge of the composition and mode of action of vaccines only arrived later, with Louis Pasteur who finally eliminated the dogma of spontaneous generation (15). The history of vaccination against infectious diseases includes a first golden era following the work of Pasteur in the 1800s followed by a second golden era from 1940 to 1970 and by the current modern era (11). Pasteur, who was one of the scientists who contributed to the derogation of the dogma of spontaneous generation (15). The history of vaccination against infectious diseases includes first a golden era, from the work of Pasteur in the 1800–1938's, a second golden age from 1940 to 1970, and then the modern era until the present (11). Pasteur established the empirical approach to vaccination, based on isolating, inactivating, and injecting the germs that cause the disease. In addition, he discovered the benefits of germ attenuation in the generation of protection and developed the anti-rabies attenuated vaccine and, together with Koch, an attenuated vaccine against anthrax bacillus, in 1881 (15).

A few years later, Diphtheria and Tetanus were discovered to be caused by bacterial toxins. With the collaboration of Gaston Ramon, in 1920, Behring described an efficacious vaccine against both diphtheria and tetanus, composed of the inactivated toxins, with alumina to improve efficacy (16). Furthermore, Albert Calmette and Camille Guerin attenuated *Mycobacterium bovis* and obtained the attenuated BCG vaccine against tuberculosis in 1924 (17). A vaccine composed by the toxoids of Diphtheria and Tetanus and inactivated *Bordetella pertussis* was only launched in the USA in 1948, but because of safety issues, a double vaccine against only Diphtheria and Tetanus continued to be used and a new acellular pertussis vaccine was only licensed in the USA in 1996 (18).

In 1930, inactivated fractionated influenza vaccines started to be produced in embryonated eggs, which are still used today.

In 1949, however, tissue culture technology enabled the production of virus vaccines against polio, measles, mumps, and rubella, and during the 1960's, against varicella zoster, rotavirus, and Influenza (2). A very effective live attenuated vaccine against yellow fever, composed of three 17D sub-strains, was developed in Cuba in the 1930s, in embryonated eggs, and has not been changed since the 1940s (19).

Under the predominance of inactivated or attenuated vaccine technology, two formulations against polio helped to reduce polio epidemics. The Salk vaccine, composed of the inactivated virus, was used on a large scale in the USA in 1954 and in 90 countries since 1959 (20). In contrast, the oral Sabin vaccine was composed of three attenuated polioviruses and started to be used worldwide as of 1962, with high adhesion and vaccine coverage. The eradication of wild type 2 poliovirus was, in fact, announced in September 2015. However, the vaccine eventually showed serious safety issues, causing reversed virulence and paralysis in immunosuppressed individuals, and promoted outbreaks of poliovirus 2 vaccine-associated paralytic poliomyelitis (21). For these reasons, in some countries, the inactivated Salk vaccine is starting to be used again.

In the 1970's, polysaccharide vaccines against pneumococcus and *Haemophilus influenzae* were launched. One decade later, the conjugation of the polysaccharides to proteins increased the antibody titers in children and allowed the induction of a T cell-mediated response (2).

Furthermore, it was only in 1982, when modern recombinant techniques enabled the production of synthetic surface antigens of the hepatitis B virus (22) and of the HPV L1-derived virus-like particles (23) that compose the HPV vaccine, which only started to be used in 2010 (2).

In summary, after live vaccination was first used against smallpox in 1796, enormous efforts were made in the early 1900s toward the development of bacterial vaccines. These efforts were successful for the control of Diphtheria, Tetanus, and pertussis. After that, the emergence of tissue culture techniques in the 1950's represented a second stage of viral vaccine development, which yielded the vaccines against polio, measles, mumps, rubella, and varicella. Since the 1970's, a third phase of research and development privileged safety and enabled the licensing of vaccines composed of molecules of microorganisms instead of the whole germs. Now, different technologies are being used, leading to polysaccharide vaccines against pneumococcus, meningococcus, and *Haemophilus influenzae*, conjugated to proteins or not, and to recombinant vaccines against hepatitis B and HPV and other organisms.

## The Delay in the Development of Protozoa Vaccines

Virus and bacterial diseases were known since the early description by Fracastoro in “De Contagione” in 1546, where the origin of diseases was attributed to small animate agents that were invisible to the naked eye (24). This theory was confirmed with the invention of microscopes by Hooke and Van Lewenhoek in 1667 and 1675, respectively (25). However, vaccines against viruses and bacteria only started to be used in 1796 and the 1800, through the work of Jenner and Pasteur, ~250 years after the first description of these pathogens (24).

In contrast, protozoan diseases such as Malaria have been known since the fifth century BCE, when Hippocrates described it as a disease associated with the bad quality of air close to swamps (mal = bad; aria = air) (26). In addition, references to Malaria have also been found in Egyptian mummies, during Roman times, and in the European Renaissance (26). However, although the first description of Malaria dates back 2500 years, no vaccine against Malaria has yet been licensed. This large delay confirms that Malaria is one of the most important neglected diseases.

Two facts might explain the delay in the development of anti-protozoal vaccines: the late descriptions of the complete biological cycles of the agents of Malaria, Chagas disease, and Leishmaniasis and of the involvement of insects in their transmission to humans. Both descriptions only occurred in the early 1900's. Ross demonstrated that mosquitoes transmit malaria parasites in 1897 (27). Carlos Chagas described how big blood-sucking insects transmit the American Trypanosomiasis, later named “Chagas Disease,” in 1908 (28). In agreement, Leishman and Donovan had described the parasite that caused visceral leishmaniasis in Indian soldiers already in 1903 (29).

Another factor is that ancient interactions between microorganisms and the immune system influenced the co-evolution of parasites and hosts which explains why it has been so difficult to develop effective vaccines against parasites (11, 30).

In addition, protozoa diseases were originally related exacerbated by poverty and the warm climates of tropical and subtropical countries. These infections raised the interest of the doctors of the Royal Army who worked in the Asian and African colonies of the British Empire. In fact, in India, Ross studied Malaria, while Leishman and Donovan described visceral leishmaniasis. Saul Adler, on the other hand, discovered the vertebrate host for *Leishmania donovani* in Palestine (9). Recently, however, global warming and the construction of new towns and roads have changed, in many countries, the ecotopes of the vectors. Hence, the incidence of the diseases is more widespread and reaches much larger geographical areas, including regions previously considered temperate, such as countries in Europe and the Americas, and also larger populations than the ones affected in earlier centuries (31).

However, Malaria and leishmaniasis are still neglected diseases and have only recently attracted the attention of vaccine manufacturers. These diseases are not endemic in the USA, where the big vaccine industry and advanced technology are concentrated. However, geopolitics is also playing a role, and as happened in the colonies of the British Empire in the past, the interest of USA companies in producing anti-protozoa vaccines increased when American troops returned home with *Leishmania* infections contracted in Middle East countries. In fact, there is also a specific support from the USA military forces in R&D for vaccines that could protect their personnel from diseases that are endemic in areas of conflict (32). However, since preventive vaccines are commercially less profitable than drug development (33, 34) most American pharmaceutical companies have focused their research on immunotherapeutic formulations (34).

## Anti-malaria Vaccines

Approximately half of the world is at risk of Malaria infection (35–37). In the poorer countries, the incidence of deaths due to Malaria is particularly high, mainly in children under 5 years old living in Sub-Saharan Africa (35). The biological cycle of the agents of Malaria, *Plasmodium falciparum, P. malariae, P. vivax, P. ovale*, and *P. knowlesi*, is highly complex. It starts when a sporozoite is injected through the Anopheles insect bite into the blood of a human host and reaches the liver, where it multiplies and produces 30–40,000 progeny inside the hepatocytes, through a pre-erythrocytic schizogony (35). When the hepatocytes rupture, the released merozoites invade red blood cells, where they rapidly multiply to produce 8–24 merozoites each (35). These merozoites are further released with massive erythrocyte rupture, which is accompanied by fever, headache, chills, and malaise (35). At this early stage of clinical signs, fever occurs periodically (24 h for *P. knowlesi*; 48 h for *P. vivax* and *P. falciparum*, and 72 h for *P. malariae*). These symptoms match the release of a new generation of merozoites into the blood. The cycles in erythrocytes are repeated, but some merozoites, instead of following the described asexual replication, begin developing into the sexual form, called gametocytes, inside red blood cells. If a mosquito bites this host, it ingests the gametocytes, which, inside the gut transform, transform into mature gametes. The female gamete can be fertilized by a masculine gamete, and the resultant ookynete can invade the gut membrane of the mosquito, where it transforms into an oocyst. Inside the oocysts, thousands of sporozoites develop, and when the oocyst is disrupted, they migrate toward the salivary gland, from where they can be forwarded into another human host during a new mosquito blood meal (35).

In spite of the fact that Malaria mortality rates have decreased recently due to interventions such as the use of insecticide nets, residence-spraying programs, and access to artemisin in combined therapy, a preventive vaccine is essential for disease control. However, no licensed vaccine has yet been made available for Malaria (35–37).

The research in Malaria vaccines started in 1930 with the use of inactivated or dead parasites. However, only partial protection was obtained in ducklings and only with the addition of adjuvants (38). This formulation was followed by studies in rodents and finally lead to the first human vaccine, which induced efficacy and was composed of killed *Plasmodium falciparum* parasites delivered by mosquito bites (39). However, since this approach was considered impractical for large-scale vaccination, synthetic peptides based on immunogenic parasite proteins started to be used for vaccination in the 1980's. The absence of clear correlates of protection for malaria was considered an important problem to overcome.

Several tests of safety and reactogenicity were needed before testing the vaccine in a Phase II trial in children of endemic areas. These tests were very laborious and time-consuming and required extensive funding, besides running the risk of negative results. To circumvent this risk, since the 1940's, controlled human Malaria infection (CHMI) has been used to test malaria vaccines and drugs and to evaluate the products in well-controlled early-phase proof-of-concept studies (35, 40). In CHMI tests, participants are inoculated with sporozoites through the bite of an Anopheles mosquito under well-controlled conditions. CHMI can therefore be used as an immunization strategy, to determine drug and vaccine efficacies, and to study the human immune response to the parasite (40). CHMI can be promoted either by inoculation of sporozoites via mosquito bite or by injection of sporozoites or *Plasmodium*-infected blood. CHMI implies that the blood-stage infection is truncated by antimalarial drug treatment. Initial tests of the RTS,S vaccine using CHMI subjects have shown its efficacy and have allowed the adjuvant and formulation to be chosen (35). Recent advances have included the use of cryopreserved aseptic non-irradiated parasites and inoculation by injection (40). However, as the current risk of multiresistant strains of *Plasmodium* is high, and as the sporozoites remain a cryptic infection, they could promote Malaria in immunocompromised subjects; therefore, it is surprising that this technique has been ethically accepted.

Furthermore, the development of synthetic vaccines against malaria started in the 1980's, and field assays were conducted in the 1990's, using the SPf66 subunit composed of three *P. falciparum* blood-stage antigens and the circumsporozoite protein (CSP) (35). The sequencing of the genome of *Plasmodium falciparum* brought hope for the design of an anti-Malaria vaccine (35). Currently, Malaria vaccines can be divided according to the developmental stage of the parasite into pre-erythrocytic vaccines (PEV), blood-stage vaccines (BSV), transmission-blocking vaccines (MSTBV) (5, 36, 39, 41), and other vaccines, such as the anti-pregnancy associated Malaria vaccines (35), anti-toxic (42), or combination vaccines (36). However, after 40 years of research and clinical trials, only the PEV vaccine has arrived at the Phase III trial stage.

The PEV vaccine protects against the early infection of hepatocytes and in this way impedes the whole parasite cycle in the host, while the BSV vaccine only blocks the blood infection and prevents the onset of the first clinical signs of disease (fever, chills, headache, and anemia). The TBV vaccines direct the immunity against the gametocyte stage that infects mosquitoes. Therefore, TBV vaccines do not impede the human infection but, instead, the antibodies raised against it, impede the binding of the gametes to the gut membranes of the insect vector blocking its development. Therefore, mosquitoes fed with the blood of a TBV-vaccinated person do not forward the parasite to the next host. That is why the vaccine is considered a transmission-blocking vaccine and, with high coverage, can block the epidemics. This is an altruistic vaccine that does not immediately protect the vaccinated subject but, at a high coverage, can interrupt the contagion and epidemics (41, 42).

The fundamental problem of the development of a Malaria vaccine is the lack of reliable correlates of protection (35, 36). There is evidence that antibodies are the main components of the immune response (37, 43), but the results of different studies are not consistent. However, epidemiological observations, IgG passive transfer studies, and experimental infections in humans all support the feasibility of developing highly effective malaria vaccines. Although 5,400 antigens have been described, the precise ones that induce protective immunity still remain uncertain (37). The urgent need for a tool for malaria prevention promoted a joint initiative of funding agencies, private companies, and international agencies (35). While the treatment of clinical cases and the use of insecticide-impregnated bed nets are already contributing to malaria control, a highly efficient vaccine is still needed.

### PEV Vaccines

One of the targets of pre-erythrocytic vaccines is the circumsporozoite (CPS) protein, which is expressed on the surface of sporozoites, is composed of 412 amino acids, and holds 37 tetrapeptide repeats and a conserved central domain (44). The anti-CPS natural and monoclonal antibodies block the sporozoite infection *in vitro* (35).

RTS,S is the leading PEV vaccine (Table 1). The early research on the RTS,S vaccine was performed with CHMI; it predicted the efficacy of the vaccine and helped to select the adjuvant and the future lyophilized formulation (45). It is formulated as a hepatitis B surface antigen (HBsAg) fused with the CPS central repeat and thrombospondin domain. The vaccine is adjuvanted with ASO1, which is a liposome composed of 3-O-desacyl-4'-monophosphoryl lipid A and the QS21 saponin from *Quillaja saponaria* Molina extract. RTS,S is therefore a recombinant complex comprising the conserved sequences of the 3D7 strain of *P. falciparum*. Its R (repeat) portion contains the conserved tetrapeptide sequence of CSP (N-acetyl neuraminic acid phosphatase, NANP) amino acid sequence repeats and the T-lymphocyte portion (T), which includes T cell epitopes separated by immunodominant CD4+ and CD8+ (Th2R and Th3R) sequences. The RT peptide is fused to the N-terminal of HBsAg, which represents the surface portion and is called “S.” Another non-fused HBsAg moiety accounts for the second “S” of RTS,S. Altogether, they comprise the RTS,S complex (35).

**Table 1:** Current licensed anti-protozoal vaccines and those in the pipeline or at the pilot scale.

**Target host**	**Target parasite**	**Vaccine**	**Formulation**	**Preventive mechanism**	**Status**
Human	*Plasmodium falciparum* pre-erythrocytic	RTS,S	CPS central repeat fused to hepatitis B surface antigen adjuvanted with ASO1 adjuvant (monophosphoryl lipid A and the QS21 saponin)	Inhibits sporozoite motility and invasion of hepatocytes	Phase III trials
Dog	*Leishmania* (L.) *infantum chagasi*	Leishmune^®^	FML antigen of *Leishmania* (L.) *donovani* and QS21 acylated and deacylated saponins	Prevents infection; blocks transmission; increases CD4+, CD8+, and CD21+ lymphocyte rates	Licensed
Dog	*Leishmania* (L.) *infantum*	Canileish^®^	LiESP/QA-21 (*L*. (L.) *infantum* Excreted Secreted Proteins (ESP) and the purified extract of *Quillaja saponaria* QA-21)	Increases Th1 IgG and IgM responses, leishmanicidal activity of macrophages, and expression of iNOS and NO	Licensed
Dog	*Leishmania* (L.) *infantum chagasi*	Leishtec^®^	Recombinant protein A2 of amastigotes of *L*. (L.) *donovani* and saponin	A2 is expressed in amastigotes and involved in resistance	Licensed
Dog	*Leishmania* (L.) *infantum*	Letifend^®^	Poly-protein Q composed of Lip2a, Lip2b, LipP0 and the H2A histone of *L*. (L.) *infantum*	Increases IgG2 and DTH response	Licensed
Human	*Leishmania* (L.) *infantum*	LeishF3	NH36-SMT antigen (Nucleoside hydrolase NH36 of *Leishmania* (L.) *donovani*, the main antigen of Leishmune^®^, the sterol 24-c-methyltransferase (SMT) from *L*. (L.) *infantum*), and glucopyranosyl lipid A-stable oil-in-water nanoemulsion	Increases the secretion of IFN-γ, TNF-α, IL-2, IL-5, and IL-10 in response to the vaccine antigen.	Phase I trials
Dogs and cats	*Giardia lamblia*	Giardiavax^®^	Inactivated trophozoites of *Giardia lamblia*	Prevention and treatment of clinical signs and reduction of cyst shedding	Licensed

Several Phase III trials were performed with the RTS,S vaccine. In the first attempt, 46% of vaccine efficacy was obtained in children, with higher efficacy in young children than in infants (46). Several efforts have been made to increase the effectiveness of the RTS,S vaccine. Combination with other antigens (47) and administration in prime-boost strategies using different vectors (48) have been tried in order to optimize the response to the antigen (35). However, important challenges still remain, such as inducing protection against all strains of the genetically diverse parasites found in nature. Evidence indicates that the RTS,S/A02 does not induce any preferential effect against any of the alleles of the CSP with sequence similarity to the 3D7 pfcsp sequence used for the vaccine preparation (49–51).

Fewer studies have been carried out on the other pre-erythrocytic vaccines: ME-TRAP (multiple epitope thrombospondin-related adhesion protein) and the whole–sporozoite vaccine. ME-TRAP is composed of B-cell, CD4+, and CD8+ T-cell epitopes of *P falciparum* liver antigens but failed to induce protection in a Phase IIb trial in children from Kenya (52). Additionally, new candidates for PEV vaccines are the highly conserved pre-erythrocytic candidate PfCelTOS (53–55) and a *Plasmodium vivax* circumsporozoite synthetic antigen called VMP-001 (54). Furthermore, going back to the first malaria vaccine, 100% protection was also obtained in six North American volunteers who were vaccinated intravenously with irradiated sporozoites. However, the route of administration makes this kind of vaccination unfeasible (35).

## RTS,S Vaccine in the Pipeline

Although the research on Malaria vaccines started in 1930, no human vaccine is currently licensed. There is, however, a prudent enthusiasm for the use of the RTS,S AS02 vaccine, which was developed by the Vaccine Initiative MVI of PATH, Glaxo Smith Kline (GSK), and several academic and research institutions (36, 52, 53). The RTS,S vaccine has been in development since 1987, and its Phase III trials were concluded in 2014. These assays were performed under Good Clinical Practice (GCP) and Good Laboratory Practice (GLP) regulations. There was a large multi-national trial enrolling 15,459 infants carried out at 11 clinical centers in seven African countries (Burkina Faso, Gabon, Malawi, Mozambique, Ghana, Tanzania, and Kenya). The RTS,S vaccine was most effective on 5 to 17-month-old children and showed a 36% reduction of severe cases of malaria (36, 54). However, this study raised a number of safety concerns (56–59). The trial was followed up for 3 and 4 years in order to check the sustained efficacy. Vaccine efficacy against severe malaria was sustained and proved after 6 or 7 years in children who received a booster. In fact, the VE (vaccine efficacy) was 36.7% in the older group (5–7 years) and 31% in younger children (3–5 years). However, VE against mild malaria was 23.7% in the older children and 15.5% in the younger group. In addition, in the highest transmission center, an increase in clinical malaria was measured in older children (VE = −30.3%). Fifteen deaths (ten girls and five boys) were recorded in the RTS,S AS/01 vaccinated children group and seven in the controls. Hence, this study concluded that RTS,S/AS01 vaccine induces protection against severe malaria. However, a gender-associated frequency of deaths was observed, which was previously detected in the open phase III trial (58). Although an increase in the incidence of uncomplicated cases of malaria was noticed in the vaccinated subjects, a delayed peak for the incidence of severe malaria was also detected. This bias trend of increasing clinical malarial cases was not expected for a Pre-erythrocytic vaccine such as RTS,S, which was designed to prevent the infection at the very early stage of liver infection. Therefore, clinical malaria was not expected to occur in vaccinated subjects. It may be argued that natural exposure to repeated malaria is needed to acquire immunity. However, chemoprevention of malaria only promoted a small increase in clinical cases (58).

A pilot study that will be performed in Malawi, Kenya, and Ghana will be used to establish the Malaria implementation Program (MVIP). This pilot study will be able to identify the feasibility of vaccination with RTS,S regarding cost-effectiveness, ethics, community dynamics, and the commitment of authorities to continuing monitoring the program (36). Much of the success of the RTS,S vaccine in Gambia was attributed to the empathic relationship between the community and the field workers (36).

Despite the enormous effort made toward testing the RTS,S vaccine in Africa and its imminent license and review for policy recommendations, the WHO considers its efficacy low and states that the global health community expects the development and licensing, by 2030, of malaria vaccines with protective efficacy of at least 75%. Hence, the available malaria vaccines are considered only as a complementary tool for the control of the disease and should not replace the current interventions (vector control, chemoprevention, diagnosis, and treatment) (59). In spite of the time, resources, and effort invested, the RTS,S Malaria vaccine that is going to be licensed is an example of how the technology used to discover and define vaccine antigens, allied with the ethical limitations of high doses of adjuvants in humans, led to a vaccine with limited efficacy, without the expected prevention and control of the disease.

### BSV Vaccines

A second scenario for Malaria vaccines is represented by the blood-stage vaccines, which prevent the disease but not the infection. Immune protection against the blood stage is mediated through neutralizing antibodies (60). Merozoite surface proteins and infected red blood cells were used as candidates for the BSV vaccines. The merozoite surface proteins MSP1, MSP2, MSP3, serine repeat antigen, erythrocyte binding antigen, ring-infected erythrocyte surface antigen (RESA), glutamate rich protein (GLURP), and apical membrane antigen 1 (AMA1) were tested as potential candidates (35). As discussed for the PEV vaccines, infection with a parasite that holds a genetic sequence different from the strain used for the BSV vaccine might determine the type of Malaria disease (35, 51, 52). Although the AMA-1 antigen recently demonstrated efficacy against clinical malaria provoked by parasites that showed genetic identity with the vaccine strain, the limitations associated with the genetic diversity of the etiological agents indicated that only a multivalent universal vaccine could be used to control Malaria (35, 61, 62). In fact, a strain-specific vaccine would not be useful considering the great diversity of strains composing the *Plasmodium falciparum* infective agent. A recent Phase III assay of the AMA-1/ASO2 formulation in Africa showed no vaccine efficacy (VE) considering the whole cohort of vaccines (VE = 17%, *P* = 0.18) but revealed allele-specific efficacy (VE = 64%, *p* = 0.03) against the vaccine strain. Hence, the AMA-1 antigen could be a component of a multivalent vaccine (54).

### TBV Vaccines

Recent concerns of malaria spreading worldwide have focused interest on prevention with transmission-blocking vaccines, which protect mosquitoes. These vaccines are designed to generate neutralizing antibodies against the antigens of the sexual-phase gametocytes or ookynetes that infect mosquitoes. TBV vaccines do not protect the vaccinated individual but, if used at high coverage, interrupt the transmission of Malaria in endemic areas (35, 39, 40, 51). TBV vaccines include assays with the P25 and P28 ookynete surface proteins (35). In contrast to the PEV and BSV vaccines, which have already been assayed in completed Phase III trials, the assays of the Pfs25 antigen are in early development and so far, have only completed Phase I trial assays, though with success. In one of them, the Pfs25 protein was engineered as a VLP, manufactured in *Nicotiana benthamiana* plants, and was formulated with Alhydrogel (63). In a second Phase I trial, Pfs25 was conjugated to the recombinant, detoxified ExoProtein A from *Pseudomonas aeruginosa* (64). The efficacy of TBV vaccines has been tested using membrane assays. In these experiments, mosquitoes are fed on chicken embryo membranes that cover small receptacles containing human blood infected with *Plasmodium*. The addition of plasma of humans vaccinated with Pfs25 to the infected blood caused a reduction of the oocytes and sporozoites found inside the guts of the insects (65). Currently, a phase II trial is evaluating the efficacy of P25-based vaccine in Malian adults (35). In comparison to PBV and BSV, there are fewer concerns about genetic diversity regarding the P-25-based vaccines (35).

## Pregnancy-Associated Malaria Vaccines

In Malaria-infected women, during pregnancy, red blood cells are retained in the placenta. Binding of *Plasmodium*-infected red blood cells is mediated by the exposure of the *Plasmodium falciparum* PfEMP1 erythrocyte membrane protein, which interacts with the chondroitin sulfate antigen of the placenta matrix (35). While this binding is responsible for the blood supply to the placenta, at the same time it increases the risk of babies with low birth weight. Currently, the research is focused on the development of a vaccine against conserved regions of the PfEMP protein in order to reduce Malaria in Sub Saharan Africa (66).

## Vaccines Against Leishmaniasis

Visceral leishmaniasis (VL) is a disease with a wide geographical distribution and with high morbidity and mortality rates, especially in less developed countries (67). Found on five continents, it is endemic in 70 countries. In 2015, Brazil, Ethiopia, India, Kenya, Somalia, South Sudan, and Sudan together accounted for 90% of all cases of the disease. Brazil concentrates 99% of the 3,500 cases reported annually in Latin America, and although the numbers remain stable, the disease is spreading toward the Southeast of the continent (68). In addition, mortality and case fatality increased in association with the expansion of the disease to new geographic areas and malnutrition (69). In 2012, the estimated global incidence of VL was between 200,000 and 400,000 new cases per year (70).

There are two epidemiological types of VL: zoonotic visceral leishmaniasis (ZVL), which is transmitted from dogs to humans, and anthroponotic visceral leishmaniasis (AVL), which is transmitted from human to human, both through an insect vector cycle. VL is the most severe form of leishmaniasis, where vital organs such as the spleen, liver, and bone marrow are affected, which involves immunosuppression and can be fatal if left untreated (31). Even among treated patients, therapeutic failure was detected in up to 10% of cases (68).

Previously, VL was considered a disease present in rural areas, but since the 1980's, it has become endemic in more urbanized regions (71) showing the clear process of epidemiological transition. The geographical spread of the disease is directly related to the migration of people from regions where VL is endemic (72). The change in the epidemiological profile is also related to anthropic actions, such as urbanization and deforestation, which cause an environmental imbalance. This confirms that environmental changes and ecological disturbances have a significant influence on disease proliferation (73).

VL is caused by *L*. (L.) *donovani* in Asia and central Africa, by *L*. (L.) *infantum* in the Mediterranean basin, Middle East, and Central Asia, and by *L*. (L.) *infantum chagasi* in South America and Central America (31, 68). VL presents symptoms of persistent systemic infection such as fever, fatigue, weakness, loss of appetite, and cachexia. Splenomegaly and/or hepatomegaly also occur, which may lead to anemia due to the persistent state of inflammation and, eventually, death. It is also characterized by progressive suppression of the cellular immune response and hypergammaglobulinemia (31, 74).

The infection caused by the species *L*. (L) *infantum chagasi* has two distinct cycles, which are defined as wild and domestic. In the wild cycle of the disease, the bush dog, the manned wolf, the field fox and the skunk are the main reservoirs. In the domestic cycle, the reservoirs are dogs. The proximity of domestic animals to humans makes them occupy a prominent position in the epidemiological chain, and dogs are considered to be mainly responsible for the persistence of VL in tropical and neotropical environments (75). The biological cycle alternates between a promastigote stage in the guts of the phlebotomine insect vectors and the amastigote stage inside the macrophages, dendritic cells, and neutrophils of the vertebrate host.

Both AVL and ZVL are lethal diseases if not treated soon after the onset of the symptoms. However, treatment by chemotherapy is highly toxic, and only a few drugs are available. In order to prevent the selection of parasite strains resistant to drugs, the chemotherapy of dogs is not recommended in Brazil. In fact, the control of VL is based on three tools: diagnosis and therapy of human patients, culling of infected dogs, and insecticide treatment of residences (31, 68). In spite of the probable success of these measures the incidence of the disease is increasing and a preventive canine and human vaccine is therefore considered to be the most suitable tool for control (76, 77).

Considering this scenario, the evolution of the vaccine development against VL was relatively rapid. While no licensed vaccine against human Malaria is currently available, in spite of being a disease described in the fifth century BCE, four licensed veterinary vaccines are available against VL, a disease known only since 1903. Besides these veterinary vaccines, there is one live human Leishmania vaccine licensed in Uzbekistan (78). In fact, a mixture of live and dead *L*. (L.) *major* parasites has been licensed for leishmanization in areas with high-risk populations. The leishmanization technique was developed by Professor Adler, and this live vaccine was exhaustively used in Israel (9, 78). Although most field studies focus on human cutaneous leishmaniasis (CL), there have been no clinical trials of first-generation vaccines using species of *Leishmania* causing VL (79).

As discussed before, dead parasites with or without adjuvants composed the first generation of vaccines against leishmaniasis. These were mainly directed toward preventing the infections caused by the agents of CL (3, 78, 80, 81). Later, second-generation vaccines were developed, which included in their formulation either genetically modified live *Leishmania* or *Leishmania* genes expressed by viruses or recombinant, synthetic, or partially purified native antigen subunits. Finally, the third-generation vaccines are composed of parasite antigens cloned in eukaryotic promoter vectors injected into the host muscle (3, 4, 80). Detailed reviews have previously summarized the vaccine antigens under research and those that were already tested in Phase II and Phase III trials (80–82), as well as the most suitable adjuvants (82). In this chapter, we will focus our review on the licensed vaccines against *Leishmania*.

Regarding the veterinary vaccines against ZVL, two preventive canine formulations have been registered in Brazil: Leishmune® in 2003 (80–84) and Leishtec® in 2008 (85). In Europe, where visceral leishmaniasis is also a canid zoonosis, two other vaccines were licensed: Canileish® in 2011 (86) and Letifend® (87) in 2016.

### Anti-leishmania Licensed Vaccines

Leishmune® was the first vaccine licensed for the prevention of canine visceral leishmaniasis (Table 1) (75, 83, 88). It is composed of the FML glycoproteic fraction of *Leishmania* (L.) *donovani* and has the QS21 *Quillaja saponaria* acylated and de-acylated saponins as adjuvants (89). The FML complex inhibited the penetration of promastigotes (90) and amastigotes (91) into macrophages in *in vitro* assays in a species-specific manner (92). In addition, FML was immunogenic for mice and rabbits, and most of the anti-FML monoclonal antibodies reacted with its 36-kDa main component (91), which was disclosed to be a Nucleoside hydrolase (NH36) phylogenetic marker of the genus *Leishmania* (93). FML also showed high sensitivity and specificity in the serological diagnosis of human (94) and dog VL (95) and can be used in ELISA assays for the control of transfusional VL (96). Furthermore, when used for vaccination, FML protected mice (97) and hamsters from *L*. (L.) *donovani* infection (84). Its main component, the antigen GP36, also promoted vaccine efficacy against VL in mice (98). The *Quillaja saponaria* (Molina) saponins were the best adjuvants for the FML-vaccine in mice (99).

Furthermore, the FML-saponin vaccine promoted strong preventive protection in a Phase II assay in mongrel dogs experimentally infected with *L*. (L.) *donovani*. The adjuvant effect of the Riedel de Haen and the QuilA saponins were compared regarding the induction of the anti-FML antibody response. The R saponin induced the antibody response more rapidly (84). Furthermore, two Phase III trials were run in a North-East Brazilian endemic area for ZVL (100, 101). In the first assay, 97% of the dogs became seropositive and 100% showed positive DTH response after complete vaccination. After 2 years, only 8% of vaccinated dogs showed mild signs of the disease, indicating that 92% protection was achieved. Among these 6 dogs, infection was confirmed by PCR analysis in only three of them. In contrast, 33% of the untreated controls developed clinical VL or died of ZVL. In fact, four control dogs died, while another six showed clinical signs, confirmed by PCR. The difference in the infection rate was highly significant, and the calculated vaccine efficacy for the FML-saponin formulation was 76% (33%−8/33% × 100 = 76%) (84, 100).

In a simultaneous field assay in North-East Brazil using FML and QuilA saponin, 100% positive antibody and DTH responses were achieved 2 months after vaccination and was sustained for at least 3.5 years (101). The infection rate was 25% (8 animals) in placebo controls and only 5% (one dog) in vaccinated animals, making a vaccine efficacy of 80%. This difference was significant and indicated that 95% of vaccinated dogs were protected. On month 41 after vaccination, the vaccinated dogs still showed a positive DTH response. No parasites were detected through microscopic observation of bone marrow smears or by PCR of bone marrow and blood samples. In contrast, PCR and microscopic observations of amastigotes were positive in three control dogs (101).

Therefore, the FML-saponin vaccine showed high efficacy against severe end-points such as severe disease and deaths due to ZVL and provided long-lasting preventive protection that was probably also responsible for the detected decrease in human VL cases in that area: from 15 cases before the beginning of the vaccination assay to zero before its end (101). Due to these results, the FML-vaccine was considered the only effective canine vaccine against leishmaniasis, in the Second–Generation Vaccines against Leishmania Meeting held by the WHO-TDR program in Mexico in 2001 (84). The FML-saponin vaccine increases the IgG2 type response compared to IgG1 antibodies, against FML in vaccinated dogs (102). This kind of response is observed in dogs vaccinated against ZVL or in naturally protected dogs (103–107). The FML-saponin vaccine, with an increased dose of the adjuvant, was also shown to be immunotherapeutic in dogs vaccinated while infected but asymptomatic (108).

In 2003, the FML-saponin vaccine was licensed to the Fort-Dodge-Pfizer-Zoetis company and started to be produced and commercialized in Brazil under the name of Leishmune®. It was the first worldwide-licensed vaccine against ZVL. The vaccine is given only to healthy and seronegative dogs. From 2004 to 2009, 550 dogs of Brazilian endemic and epidemic areas in Minas Gerais and São Paulo states were vaccinated with Leishmune® (83). This trial reported that Leishmune® kept the immunogenicity and efficacy potential previously described for the laboratory formulation. While 39% deaths and 20.6% symptomatic cases were detected among untreated dogs, the group vaccinated with Leishmune® showed only 1% deaths and 1.2% symptomatic cases (83). Leishmune® has been shown to be safe in dogs of the same cohort (109). Besides their excellent adjuvant potential, QS21 saponins induce strong inflammatory responses with some adverse effects (AE) (110). The most frequent systemic AEs observed in humans were headache, fatigue, insomnia, pyrexia, nausea, diarrhea, dizziness, anxiety, and back pain. Hematoma and injection site pruritus were also reported. To reduce AE, the QS21 saponin was formulated inside vesicles (ISCOM, ISCOMATRIX, or Matrix-M™) (110). The safety evaluation of Leishmune®, however, disclosed only transient reactions of local pain (40.87%), anorexia (20.48%), and apathy (24.17%) (109). Local swelling reactions, vomit, and diarrhea were only detected in 15.90, 2.4, and 1.5% of the individuals, respectively. All effects showed significant declines from the first to the third dose and were mild. Adult dogs developed smaller swelling reactions than puppies, indicating that they had more resistance to the inflammatory response promoted by the saponins. No death due to anaphylaxis occurred, and allergic reactions were noted only in 0.1% and transient alopecia at the injection site in 0.28% of the injected dogs. All the mild adverse events in response to Leishmune injection were transient and disappeared before the injection of the following vaccine dose, confirming the tolerability of the vaccine (109). Besides the increase in antibodies and IDR responses, Leishmune® enhanced the frequencies of CD8+ T cells, as was expected for the use of *Quillaja saponaria* saponins (109), and of CD21+ B cells and sustained levels of CD4+ T cells (83, 108). Leishmune® was also immunotherapeutic in naturally (108, 111) and experimentally infected dogs (112). There were no deaths due to ZVL 22 months after complete immunotherapy, and 90% of the dogs were asymptomatic, healthy, and parasite-free. In contrast, 37% of infected untreated control dogs died of ZVL (108). In addition, Leishmune® promoted the clinical and parasitological cure of ZVL and reduced the deaths (111), while in combination with Amphotericin, it additionally promoted negative results in PCR, thus determining the sterile cure (111).

Furthermore, seronegative dogs vaccinated with Leishmune® and exposed to phlebotomines in a highly epidemic area showed a lack of *Leishmania* antigen in their skins and *Leishmania* DNA in their blood and lymph nodes. In contrast, 25, 15.7, and 56.7% of positivity was recorded in the same respective variables in untreated control dogs (113), indicating that Leishmune®-vaccinated dogs did not expose the parasite to the insect vector in endemic areas. Furthermore, Leishmune® proved to be a TBV vaccine (114), as described previously for the anti-Malaria vaccine (41, 42). In fact, anti-FML antibodies raised by Leishmune® in dogs prevent the binding of the parasite to the phlebotomine insect guts *in vitro* and the development of promastigotes in the guts of colony-reared phlebotomines *in vivo*. Sandflies were fed human blood, parasites, and sera. Insects that ingested anti-FML antibodies showed 74% fewer parasites in their guts than insects that fed upon sera of pre-immune dogs (114). In contrast, sera of naturally infected dogs promoted a 431% increase in the parasite load observed in insect guts. Therefore, Leishmune® is not only capable of reducing the exposition of parasites in vaccinated dogs but also induces a robust antibody response that curtails the transmission of the disease in nature (84, 113, 114).

These summarized results explain why, when used on a large scale, in Brazilian endemic areas, Leishmune® decreased the incidence of human and canine ZVL (77). If Leishmune® reduces the presence of parasites in dog skins (113) and is a transmission-blocking vaccine (114), its use in dog vaccinations would reduce the canine and, subsequently, the human cases of the disease, interrupting epidemics. The impact of the use of Leishmune® on the official epidemiological data of Campo Grande, Araçatuba, and Belo Horizonte, three important Brazilian towns where ZVL is endemic, was analyzed. The results showed that, in spite of promoting an increase in anti-FML antibodies, vaccination with Leishmune® did not interfere with a serological control campaign that screened 110,000 dogs. Positivity was detected only in 1.3% of the animals, 76 among 5,860 dogs, in which the presence of parasites could not be detected (77). Furthermore, the potential additive effect of vaccination with Leishmune® over dog culling from 2004 to 2006 was studied. In Araçatuba, decreases of 25% in dog cases and 61% in human cases were reported. In Belo Horizonte, rising numbers of canine and human cases were observed in the districts where no vaccination was performed, while the vaccinated district exhibited stable or decreased incidences after Leishmune® vaccination. The districts that had the highest vaccination rates (63.27 and 27.27%, respectively) exhibited the strongest decrease in human incidence (−36.5%) and the lowest dog incidence (−3.36 and 1.89%, respectively). In contrast, the almost unvaccinated districts showed a very high increase of canine incidence (24.48, 21.85, and 328.57%) and human incidence of VL (14, 4, and 17%, respectively) after the two year study period. Therefore, the decline of canine and human incidences is correlated with the increase in the number of vaccinated dogs (77). The results of the above studies confirmed Dye's predictions (76) and proved the beneficial contribution of Leishmune® vaccination over dog culling in the control campaign (77). In fact, Leishmune® became a new important tool to be used in the preventive epidemiological control of VL.

The second vaccine licensed for ZVL was Leishtec®, which is composed of the recombinant protein A2 of amastigotes of *L*. (L.) *donovani* and saponin. A2 is an important virulence factor, preferentially expressed in amastigotes, that is involved in resistance in the visceral organs and in resistance against stress (115, 116). A2 was shown to be protective against VL in mice (117–119), in non-human primate models (120), and in a kennel assay with beagle dogs (85).

Additionally, Leishtec® was the second vaccine licensed for commercialization in Brazil, in 2008 (Table 1). Remarkably, the vaccine was licensed without any information about efficacy in field assays. In fact, the first Phase III trial assay of Leishtec® was only published in 2016 (121). Moreover, the experiment was performed in a Brazilian endemic area with low infective pressure. Accordingly, the end-points of efficacy were not severe disease or deaths in canine visceral leishmaniasis, as were used for evaluation of Leishmune® previously (100, 101), but instead, seropositivity confirmed by parasitological assays alone or in combination with xenodiagnosis (121). Considering these less severe standards and based on the parasitologically positive cases, the vaccine efficacy of Leishtec® was 71.4%. When parasitologically and xenodiagnosis-positive cases were considered, vaccine efficacy was 58.1% (121). While the first field trial of Leishtec® was performed in a low infective pressure area, a second Phase III trial was run in a high transmission rate area, and the vaccine efficacy was only 35.7% (122). In fact, the incidence of canine VL was 42% in controls and 27% in vaccinated dogs (122). Furthermore, 43% of the vaccinated dogs developed the disease over time, and seroconversion was higher in vaccinated dogs than in controls (122).

Therefore, in contrast to Leishmune®, which showed 76–80% efficacy based on strict end-points (deaths and clinical cases) (100, 101) and promoted the decrease of canine and human incidence of VL in areas of high infective pressure (77), the second field assay of Leishtec® showed that, when combined with dog culling, Leishtec® may not reduce the canine incidence of leishmaniasis in areas of high transmission and may have no impact on the human incidence of the disease (122). A recent study on owned hunting dogs infected with *L*. (L.) *infantum* showed that, if used for immunotherapy, Leishtec® gave a 25% reduction in the risk of progression to clinical symptoms and a 70% reduction in mortality (123). In contrast, a higher potency was described for Leishmune®, which, when used for immunotherapy, gave an 80% reduction in symptomatic cases, a 100% reduction in parasite-positive cases, and a 100% reduction in deaths, and, when used in combination with chemotherapy, also promoted a sterile cure with negative PCR results (111).

This evidence explains why many comparatives studies disclosed results of higher efficacy for Leishmune® than for Leishtec® and/or LBsap vaccines (124, 125) as well as a stronger induction of the immune and pro-inflammatory response (126–128) and enhancement of the proportions of CD8^+^ T cells secreting-IFN-γ (129).

It was only in 2011 that the first European anti-*Leishmania* vaccine was licensed to Virbac, under the name of CaniLeish® (Table 1). The vaccine is composed of the *Leishmania (L.) infantum* Excreted Secreted Proteins (ESP) and the purified extract of *Quillaja saponaria* (QA-21). Canileish® promotes a Th1 predominant immune response that lasts for a full year (130) and induces an increase in IgG and IgM immunoglobulins in sera after the third dose, which is probably due to the induction of a pro-inflammatory response (131). A Phase III trial performed in two highly endemic areas of the Mediterranean basin, one near Naples and the other near Barcelona, used PCR and parasite cultures to confirm infection (86). In this assay, 63% of vaccine efficacy was observed on month 24 after vaccination if active infection is considered as the main end-point. Considering the VL symptoms, the vaccine efficacy was 68.4% and the protection rate was 92.7% (86). Accordingly, it has been admitted that there are only two licensed vaccines that are able to confer significant protection against the most severe endpoints: disease and death under natural conditions. One is the FML-saponin (Leishmune®) vaccine in Brazil, and the other is the LiESP/QA-21 (CaniLeish®) vaccine in Europe (132). Both Leishmune® and Canileish® are composed of purified parasite complex fractions and both are adjuvanted by *Quillaja saponaria* Molina adjuvants (132). In addition, Canileish® induces leishmanicidal activity in macrophages and increases the iNOS and NO expressions, which finally kills the parasite (133).

Finally, the fourth licensed vaccine against canine VL, Letifend® (Table 1), is composed of the poly-protein Q, containing five genetically fused antigenic determinants from the Lip2a, Lip2b, P0 acidic ribosome proteins, and H2A histone of *L*. (L.) *infantum*. Letifend® does not contain adjuvant (87, 134) and was licensed in Europe in 2016. This vaccine protected dogs from experimental infection (135). In fact, vaccinated dogs showed a lower number and lower intensity of symptoms than controls and no signs of parasites in target organs (135). Furthermore, Letifend® was assayed in Phase III trials in France and Spain, where cases were represented by dogs that showed clinical signs confirmed by positive serological assay and that held parasites in bone marrow or lymph nodes, as evidenced by PCR or microscopic analysis of smears. In these assays, the vaccine efficacy disclosed for Letifend® was 72% (87).

### Perspectives on Human Vaccination Against VL

Although different degrees of prevention of canine leishmaniasis are currently achievable, depending on which of the four veterinary licensed vaccines is used, a subsequent decrease of human incidence of VL was only described in areas where dogs were vaccinated with Leishmune® (77). Meanwhile, direct protection obtained through human vaccination is not yet possible, since no human vaccine has achieved the status of registration. However, two human vaccines have been tested in clinical trials. The first-generation Alum-precipitated autoclaved *Leishmania* (L.) *major* vaccine was assayed with BCG in a phase II study with children in Sudan; it showed to be immunogenic and safe and converted the leishmanin skin test to positive (136). The dosage of the vaccine was also studied (137). Furthermore, safety and immunogenicity were also observed in 76% of the human volunteers that produced IFN-γ in response to the *Leishmania* lysate (138). However, the vaccine only induced 43% efficacy among vaccinated subjects in whom the intradermal skin reaction was converted (79). This performance was expected for a first-generation vaccine against leishmaniasis (3).

The second human vaccine that was tested in clinical trials is LeishF3 (Table 1), composed of the Nucleoside hydrolase NH36 of *Leishmania* (L.) *donovani* (93, 139–145), which is the main antigen of the Leishmune® vaccine (91, 94, 139), and the sterol 24-c-methyltransferase (SMT) from *L*. (L.) *infantum*. The vaccine is adjuvanted by glucopyranosyl lipid A-stable oil-in-water nanoemulsion (GLA-SE) (146, 147). The NH36 was first described in 1993–1996 as the main antigen of the FML complex, against which the antibodies of human patients with VL and anti-FML monoclonal antibodies react (91, 94). Antigen-specific responses of IL-2 and TNF-α were generated in Balb/c and C57 Bl6 mice vaccinated against visceral and cutaneous leishmaniasis (140, 142–144) and in dogs treated against ZVL (148). In addition, the generation of Th1 and Th17 responses against the domains of NH36 was observed in human asymptomatic and cured *L*. (L.) *infantum chagasi*-infected subjects from Brazil (145) and *L*. (L.) *infantum*-infected individuals from Spain (149).

Recently, the NH36-SMT-GLA-SE vaccine (Leish-F3) was tested in a phase I trial in healthy volunteers in the USA, in a non-endemic area for VL (147). Increased secretion of IFN-γ, TNF-α, IL-2, IL-5, and IL-10 cytokines was detected in response to the vaccine antigen (146). Additionally, a strong cytokine response to each component of the vaccine was also found in VL patients from Bangladesh. They exhibited CD4 Th1 cell responses to NH36 and SMT, with secretion of IFN-γ, TNF, and IL-2 (146) and also IL-5 and IL-10 in whole blood assays. In contrast, low levels of IL-5 and IL-10 and sustained levels of IL-2 (against NH36 and SMT) and TNF-α (against NH36) were found in vaccinated mice, in which the parasite load was reduced (146, 147). The vaccine was finally considered safe and well-tolerated in human volunteers (146). In fact, the NH36-SMT vaccine (LEISH-F3) is the first human subunit vaccine against VL that has reached the Phase I clinical trial stage (147), although no human vaccine has yet been licensed against the disease.

### Anti-giardia Vaccine

*G. lamblia* is an enteric protozoan parasite that causes endemic enteric infections of human and animal clinical importance. It is one of the most common causes of enteric infections in children and in farm and domestic animals (dogs and cats) and is mainly associated with poor sanitary conditions and zoonotic transmission (150). Giardiasis is a common cause of waterborne outbreaks of diarrhea due to the transmission of *Giardia* cysts. It is a zoonotic disease. Clinical manifestations of giardiasis include diarrhea, loss of weight, anorexia, and lethargy, both in humans and animals. Currently, there is no human-licensed vaccine against giardiasis, but a crude veterinary vaccine composed of the total lysate of *Giardia lamblia* trophozoites, called GiardiaVax® (Table 1), was licensed as a preventive tool (151). It was shown to attenuate giardiasis symptoms and to prevent cysts spreading in cats and dogs (151) but not in calves (152).

## Conclusions

Prevention of severe diseases by inoculation or contagion with the respective germ is an intuitive notion and has been practiced since pre-historical times in order to generate a protective immune response in healthy persons or animals. Vaccination, in this way, contributed to the health and survival of even illiterate communities. In fact, vaccination did not strictly start with academic knowledge. Different kinds of vaccinations were practiced to avoid diseases since 1796 in Europe and since the 1500's in Africa, China, and India. However, viral and bacterial diseases started to be controlled by vaccination 250 years after the invention of the microscope and the description of most microorganisms. One exception is the vaccine against Variola, which is a viral disease known since the 16th BCE that was successfully eradicated through massive international vaccination. In contrast, Malaria, a severe and lethal protozoan disease, has been known since the fifth century BCE, but no vaccine for it has yet been granted a license.

Vaccination started with the use of live germs and evolved through the use of germs of related species that do not cause disease but instead provide heterologous protection in the host. After that, inactivation of germs contributed to an increase in safety. More recently, subunit native or recombinant DNA and synthetic safe vaccines have been developed thanks to advances in biotechnology and industrial development. In this way, vaccines gained safety but lost immunogenicity and efficacy. Only in some cases has this effect been reverted by the use of potent adjuvants. Our historical approach leads us to conclude that the vaccines that were not developed using live, heterologous, or attenuated formulations in the period ranging from 1600 to the 1800's faced so many regulatory limitations and safety concerns in modern times that they lost their capability to control the epidemics. This seems to be the case of the human Malaria vaccine. The first candidate that applied for a licensing status is not a live vaccine but is, instead, a modern recombinant, very defined formulation that, unfortunately, shows a very low vaccine efficacy. Therefore, protozoan infections are the cause of neglected diseases against which human vaccines have been developed only recently using the most modern regulatory and safety criteria. The situation is better in the case of visceral leishmaniasis, which is a canid zoonosis. The licensed second-generation vaccines against canine VL show higher efficacies than the human Malaria vaccine. As they are veterinary formulations, they can include higher concentrations of antigen and adjuvants, which enable them to control veterinary epidemics and indirectly, the incidence of the human infection as well.

## Author Contributions

CP-S designed this review. CP-S and DN wrote this paper and agreed on its submission.

### Conflict of Interest

CP-S and DN are two of the inventors of the patent file PI1015788-3 (INPI Brazil). CP-S is the inventor of the patent US 13/383,952 and one of the inventors of the patents EP1893640B1, all of which are owned by the Federal University of Rio de Janeiro.
